# Expert consensus on the use of systemic glucocorticoids for managing eosinophil-related diseases

**DOI:** 10.3389/fimmu.2023.1310211

**Published:** 2024-01-05

**Authors:** Victoria del Pozo, Irina Bobolea, Manuel J. Rial, Georgina Espigol-Frigolé, Roser Solans Laqué, Jesús María Hernández-Rivas, Elvira Mora, Astrid Crespo-Lessmann, José Luis Izquierdo Alonso, María Sandra Domínguez Sosa, Juan Maza-Solano, Belén Atienza-Mateo, David Bañas-Conejero, Abraham L. Moure, Íñigo Rúa-Figueroa

**Affiliations:** ^1^ Immunology Department, Instituto de Investigación Sanitaria Fundación Jiménez Díaz (IIS-FJD), Madrid, Spain; ^2^ Allergy Department, Severe Asthma Unit, Hospital Clínic Barcelona, Barcelona, Spain; ^3^ Allergy Department, Severe Asthma Unit, Complexo Hospitalario Universitario A Coruña, A Coruña, Spain; ^4^ Centro de Investigación Biomédica en Red de Enfermedades Respiratorias (CIBERES), A Coruña, Spain; ^5^ Department of Autoimmune Diseases, Hospital Clinic Clínic, University of Barcelona, Institut d’Investigacions Biomèdiques August Pi i Sunyer (IDIBAPS), Barcelona, Spain; ^6^ Autoimmune Systemic Diseases Unit, Internal Medicine Department, Vall d’Hebron Hospital, Autonomous University of Barcelona, Barcelona, Spain; ^7^ Department of Medicine, University of Salamanca & Servicio de Hematología, Hospital Universitario de Salamanca, Instituto de Investigación Biomédica de Salamanca (IBSAL), Salamanca, Spain; ^8^ Hematology Department, La Fe University and Polytechnic Hospital, La Fe Research Institute, Valencia, Spain; ^9^ Department of Respiratory Medicine, Hospital de la Santa Creu i Sant Pau, Universitat Autònoma de Barcelona, Barcelona, Spain; ^10^ Department of Medicine and Medical Specialties, University of Alcalá, Alcalá de Henares, Madrid, Spain; ^11^ Pulmonology Service, Guadalajara University Hospital, Guadalajara, Spain; ^12^ Rhinology Unit, Department of Otolaryngology, Head and Neck Surgery, University Hospital of Gran Canaria Dr. Negrín, Las Palmas de Gran Canaria, Spain; ^13^ Rhinology Unit, Department of Otolaryngology, Head and Neck Surgery, Virgen Macarena University Hospital, Sevilla, Spain; ^14^ Division of Rheumatology, University Hospital of Marqués de Valdecilla, Instituto de Investigación Marqués de Valdecilla (IDIVAL), Immunopathology group, Santander, Spain; ^15^ Specialty Care Medical Department, GSK, Madrid, Spain; ^16^ Rheumatology Department, University Hospital of Gran Canaria Dr. Negrín, Las Palmas de Gran Canaria, Spain

**Keywords:** adverse events, eosinophilic diseases, systemic glucocorticoids, biologics, tapering, treatment optimisation

## Abstract

Eosinophil-related diseases represent a group of pathologic conditions with highly heterogeneous clinical presentation and symptoms ranging from mild to critical. Both systemic and localized forms of disease are typically treated with glucocorticoids. The approval of novel biologic therapies targeting the interleukin-5 pathway can help reduce the use of systemic glucocorticoids (SGC) in eosinophilic diseases and reduce the risk of SGC-related adverse effects (AEs). In this article, a panel of experts from different medical specialties reviewed current evidence on the use of SGC in two systemic eosinophilic diseases: Eosinophilic Granulomatosis with PolyAngiitis (EGPA) and HyperEosinophilic Syndrome (HES); and in two single-organ (respiratory) eosinophilic diseases: Chronic RhinoSinusitis with Nasal Polyps (CRSwNP) and Severe Asthma with Eosinophil Phenotype (SA-EP), and contrasted it with their experience in clinical practice. Using nominal group technique, they reached consensus on key aspects related to the dose and tapering of SGC as well as on the initiation of biologics as SGC-sparing agents. Early treatment with biologics could help prevent AEs associated with medium and long-term use of SGC.

## 1 Introduction

Eosinophilic diseases are characterized by the presence of high levels of eosinophils in the blood (eosinophilia) and/or in certain tissues, such as respiratory or digestive systems or in the connective tissue, where they can cause inflammation and organ damage (1). The most common causes of eosinophilia or localized eosinophil tissue infiltration are allergic reactions, parasitic infections and certain malignancies, such as Hodgkin lymphoma and leukemia (2, 3).

The clinical presentation of eosinophilic diseases is highly heterogeneous, ranging from localized eosinophilic disease in which a single organ is involved, such as the lung, to systemic disease in which multiple organs are affected, as occurs in Eosinophilic Granulomatosis with PolyAngiitis (EGPA). The magnitude of eosinophilia also varies widely.

Treatment depends on the cause of eosinophilia and the organs and/or systems involved, although it has traditionally relied on eosinophil attenuation by using local and systemic glucocorticoids (SGC) (4). Various international scientific societies have issued guidelines to aid the management of different eosinophilic diseases and the use of SGC (5–11). Prednisone is the most commonly prescribed glucocorticoid as first-line therapy for patients with systemic or localized eosinophilic diseases (12, 13). There is considerable variation in the dose of SGC used and duration of treatment, regardless of whether the disease is localized or systemic (5, 7, 14, 15). In both cases, because SGC are often used in high doses and/or over prolonged periods, patients are at increased risk of well-known SGC-related adverse effects (AEs), such as serious infection (16), and corticoresistance (17–19).

Several novel biologic therapies targeting interleukin-5 (IL-5) and the IL-5 receptor have recently been approved for clinical use, and can help reduce the dose or avoid the use of SGC in eosinophil-related diseases (3, 20–22).

For many eosinophil-related diseases, there is no universally accepted modality of SGC regimen (14, 15, 23, 24). Because of the lack of evidence for some pathologies, healthcare professionals often have to extrapolate the evidence available in managing one pathology, such as Severe Asthma with Eosinophil Phenotype (SA-EP) for which there is more evidence on SGC tapering, to another, such as EGPA and HyperEosinophilic Syndrome (HES), where the scarcity of patients makes this evidence more difficult to generate.

In this article, a panel of experts from different medical specialties reviewed current evidence on the use of SGC in two systemic eosinophilic diseases: EGPA and HES, and in two single-organ (respiratory) eosinophilic diseases: Chronic RhinoSinusitis with Nasal Polyps (CRSwNP) and SA-EP (**Box 1**); and contrasted it with their experience in clinical practice. They reached consensus on key aspects of the management of these diseases with SGC and on when to initiate biologics with the purpose of reducing the risk of SGC-associated AEs. Further research into the management of eosinophil-related diseases with biologics will help to establish specific evidence-based guidelines to minimize SGC-related AEs and optimize therapy.

Box 1 **Eosinophil-related diseases**

**Systemic eosinophilic diseases:**

**Eosinophilic Granulomatosis with PolyAngiitis (EGPA)**

EGPA, formerly called Churg-Strauss syndrome, is a rare form of vasculitis that primarily affects small blood vessels. Individuals diagnosed with EGPA usually have a history of asthma or allergies.

EGPA is a chronic illness with cycles of relapse and remission that can cause serious health problems.

Treatment typically includes OCS used in combination with non-glucocorticoid immunosuppressive agents. In 2017, mepolizumab (Nucala) was the first biologic drug to be approved by the U.S. Food and Drug Administration (FDA) for the treatment of EGPA in adults (25).

**HyperEosinophilic Syndrome (HES)**

HES is the term used to refer to a group of rare blood disorders characterised by very high numbers of eosinophils and end-organ damage.

People with HES usually have more than 1,500 eosinophils/microliter in their blood for 6 months or more (vs less than 500), and the cause cannot be identified. These eosinophils make their way into various tissues, causing inflammation and eventually organ dysfunction. The most commonly involved organs in HES include the skin, lungs, heart, gastrointestinal tract, and nervous system.

Patients with HES often keep using SGC to reduce blood eosinophil count despite the fact that new targeted therapies against eosinophils (i.e. anti-IL-5 biologics) have been approved for this indication in Europe and the US (26–28).

**Localised (respiratory) eosinophilic diseases:**

**Severe Asthma with Eosinophil Phenotype (SA-EP) **

SA-EPA is a rare type of asthma that is usually only found in adults ages 35-50 with no allergies. Patients can struggle to manage their symptoms even with high doses of inhaled corticosteroids.

Short cycles of high-dose OCS are used to reduce the frequency of eosinophilic asthma attacks. Several biologics, including benralizumab, mepolizumab and reslizumab have been approved for treating SEA (29).

**Chronic RhinoSinusitis with Nasal Polyps (CRSwNP) **

CRSwNP is a chronic inflammatory disease of the nasal mucosa and paranasal sinuses. Patients suffer from persistent nasal congestion, rhinorrhoea and loss of smell. CRSwNP has a substantial impact on patients’ HRQoL, including sleep quality (30).

Up to 30% of patients with CRS have nasal polyps. The symptoms of CRSwNP are uncontrolled by current standards of care in one-third of patients, especially in cases associated with high levels of eosinophils such as aspirin-exacerbated respiratory disease (31).

Sinonasal surgeries and/or corticosteroids are the most common treatments for CRSwNP. Biologics are an emerging treatment option for patients with severe uncontrolled CRSwNP.

## 2 Methods

The authors, a multidisciplinary panel of expert clinical immunologists, pulmonologists, ENT (ear, nose and throat) specialists, allergists, rheumatologists, hematologists, and internal medicine specialists involved in the management of eosinophilic diseases across Spain, reviewed 20 articles on severe asthma, 14 articles of nasal polyposis, 10 articles on HES and 12 articles on EGPA. These articles were selected after a PICO-based search (P, patients with eosinophilic disease; I, treatment with systemic corticosteroids; C, without treatment with systemic corticosteroids; O, dose and/or time until the development of AEs) using the PubMed database and predefined keywords (oral glucocorticoids, systemic glucocorticoids, corticosteroids, eosinophils, eosinophilic diseases, chronic rhinosinusitis, nasal polyps, polyposis, severe asthma, hypereosinophilic syndrome, eosinophilic granulomatosis with polyangiitis, Churg-Strauss syndrome). Only articles published in English between 2007-2022 were considered. Search results were filtered to select evidence relating to: current recommendations on the use of SGC for treating eosinophil-related diseases; the impact of SGC at the respiratory tract and systemic level; maximum acceptable SGC dosing; and SGC sparing and AE prevention strategies.

At a meeting held on the 19^th^ of September 2022 in Madrid, Spain, the panelists contrasted the gathered evidence on the use of SGC in eosinophilic diseases (focusing on CRSwNP, SA-EP, EGPA and HES) with their experience in managing these diseases in daily clinical practice. Using nominal group technique, they reached consensus on key aspects related to the dose and tapering of SGC, as well as on the initiation of biologics as SGC-sparing agents, with the aim of reducing the risk of SGC-related AEs in the different eosinophil-related diseases discussed.

## 3 Results

### 3.1 SGC dosing and tapering for eosinophilic diseases

In systemic diseases such as EGPA or HES, it is still common practice to start with the maximum dose of SGC to induce remission and then decrease it (EGPA, up to 1mg/kg/day, maximum 80 mg/day prednisone or equivalent; HES, up to 1mg/kg/day prednisone or equivalent, see **Table 1**). For respiratory eosinophilic diseases like CRSwNP and SA-EP, SGC are used as a last resort for exacerbations in uncontrolled patients and withdrawn without tapering.

**Table 1:** Suggested SGC use for eosinophil-related diseases from selected learned societies and groups of experts.

	Systemic eosinophilic diseases:	Induction therapy (adults)	Tapering	Maintenance dose	Alternative treatment strategy	REF
**EGPA**	EGPA Consensus Task Force	1mg/kg/day for 2–3 weeks, max 60mg/day	to 20mg/day after 3 months and to 10mg/day after 6 months. No universally accepted protocol	<7.5mg/day	Combination with non-glucocorticoid immunosuppressive agent	Groh M, et al. 2015 (5).
	European League Against Rheumatism (EULAR) recommendations	60mg/day, 2 weeks	7.5–10mg is desirable by 3 months	7.5–10mg is desirable by 3 months. Remission-maintenance therapy to be continued for at least 24 months following induction of sustained remission. Early cessation of therapy is associated with an increased risk of relapse.	Active, severe EGPA: cyclophosphamide or rituximab with glucocorticoid. Rapidly progressive or pulmonary haemorrhage: plasma exchange. Non organ threatening: methotrexate or mycophenolate mofetil with glucocorticoid.	Yates M, et al. 2016 (6).
	American College of Rheumatology/Vasculitis Foundation Guideline	Up to 80mg/day (IV pulse or high-dose daily oral glucocorticoid).	ND	The duration of glucocorticoid therapy should be guided by the patient’s clinical condition, preferences, and values. Typically ≥18 months	In active, severe EGPA: glucocorticoids should be combined with a non-glucocorticoid immunosuppressive agent such as cyclophosphamide or rituximab	Chung SA, et al. 2021 (7).
**HES**	WHO	Prednisone 1mg/kg. Retrospective analyses show median starting dose between 30-40 mg/day for 4 weeks.	ND	Median maintenance dose ranges between 5-60mg daily. Duration 2-20 years	Relapse, signs of organ damage, and/or significant increase of the eosinophil count with a prednisone dose > 10mg daily is an indication for the addition of other agents. Second line-agent in HES after steroid failures, interferon-α (IFN-α). Mepolizumab was recently FDA-approved for patients with idiopathic HES and has shown efficacy in decreasing the risk of flares and as a steroid-sparing therapy.	Shomali WG, et al. 2022 (8).
	Localised eosinophilic diseases:	Induction therapy (adults)	Tapering	Maintenance dose	Alternative treatment strategy	REF
**CRSwNP**	The European Position Paper on Rhinosinusitis and Nasal Polyps 2020	1-2 courses of 7-21 days per year and topical nasal corticosteroids. Doses range between 25-60mg/day. Duration of effect approx 12 weeks	Withdraw	None	In patients with bilateral polyps who have undergone surgery or who are unfit for surgery, at least three of the following criteria should be met for starting a biological: Evidence of type 2 disease; need for at least two courses of SGC per year or continuous use (>3 months) of low-dose SGC or contraindication to SGC; significantly impaired quality of life (SNOT-22 ≥40); anosmia on smell test; diagnosis of comorbid asthma	Fokkens WJ, et al. 2020 (9).
	British Society for Allergy and Clinical Immunology recommendations	0.5mg/kg for 5-10 days and topical nasal corticosteroid	Withdraw	None	SGC should be used briefly and always in combination with a topical nasal corticosteroid. Surgical intervention should be reserved for treatment failures	Scadding GK, et al. 2008 (10).
**SA-EP**	GINA	40-50mg/day usually for 5–7days (adults)	Can be stopped without tapering.	≤ 7.5mg/day. SGS maintenance should be considered as a last resort.	For patients with difficult-to-treat asthma and blood eosinophils ≥300/μl, investigate for non-asthma causes including testing for Strongyloides infection before considering biologic therapy. For patients with hypereosinophilia, causes such as EGPA should be considered and anti-IL4R is preferably avoided as such patients were excluded from the Phase III studies	Global Initiative for Asthma. 2022 (32).

However, the patterns of SGC use differ widely between specialists and the different eosinophilic diseases. This is partly due to different guideline recommendations, limited evidence for some eosinophilic diseases and to the individual patient’s response to SGC and risk of AEs.

The experts agreed with previous literature that in localized diseases, a cumulative dose exceeding 1 g/year prednisone or equivalent is indicative of poor control and is associated with SGC-related AEs (33, 34), but they are aware that in clinical practice this dose is exceeded in many patients. Data from the Optimum Patient Care Research Database (OPCRD) and the British Thoracic Society (BTS) Difficult Asthma Registry show that the median cumulative SGC dose over the 2 study years was 3920 mg in severe asthma patients and that 93% of patients had one or more AE linked to SGC exposure (35). There is growing evidence in the literature that even very brief cycles of SGC have a cumulative effect that significantly increases the risk of developing SGC-related AEs, including osteoporosis, pneumonia, cardiovascular diseases, hypertension, glaucoma, depression/anxiety, type 2 diabetes and growth retardation in children (34, 36–39).

#### 3.1.1 Considerations for systemic eosinophilic diseases: EGPA and HES

To control disease flares in patients with systemic eosinophilic diseases the experts would prescribe between 30-60 mg/day prednisone or equivalent (or 0.5-1 mg/kg/day) for 14–21 days. The prescribed dose can vary widely as it depends on type of disease, symptom severity, the patient’s history and existing medication. After an initial course, the dose should be reduced; rapidly at first (halved after 2–4 weeks) and then more slowly over the course of 3–6 months until withdrawal or a minimum maintenance dose, < 5 mg/day, is achieved, in agreement with the latest recommendations from the European Alliance of Associations for Rheumatology (40). In severe EGPA cases, intravenous pulses of methylprednisolone are frequently used.

The maintenance dose threshold to prevent the development of serious AEs in patients with systemic eosinophilic diseases is difficult to establish given the heterogeneity of patients and the length of exposure, which determines cumulative dose. The risk/benefit balance of low dose SGC in systemic rheumatic diseases is still unclear (41), but the experts agreed that the lowest effective dose of SGC should be used for the shortest time possible.

In EGPA, the duration of corticosteroid therapy should be guided by the patient’s clinical condition, values and preferences. There is insufficient published evidence to support a specific duration of SGC and there are discrepancies between guidelines (12, 42). Many patients with EGPA require low dose glucocorticoids to control their asthma and other symptoms. A reduced-dose regimen has been shown to decrease the risk of serious infections and minimize SGC toxicity (7, 43).

Patients with HES often keep using SGC to reduce blood eosinophil count despite the fact that new targeted therapies against eosinophils (i.e. anti-IL-5 biologics) have been approved for this indication in Europe and the US (26–28).

#### 3.1.2 Considerations for respiratory eosinophilic diseases: CRSwNP and SA-EP

SGC use in CRSwNP and asthma is restricted by guidelines for the management of exacerbations and as a last resort when no other treatment options are available. During worsening of symptoms, the experts would prescribe a 7–21 day cycle of SGC for adults (40–50 mg of prednisone or equivalent per day, depending on patient’s weight) (39, 44–46). If the patient responds, treatment can be discontinued without tapering. As the risk of serious AEs increases with the number of cycles and cumulative dose, it was agreed that these patients should not be prescribed more than 2 cycles/year (38).

Additional research is still needed to determine the minimal effective dose and duration of SGC therapy for the treatment of CRSwNP to prevent the development of AEs (23, 47). Despite evidence that short courses of SGC can ameliorate the symptoms and reduce polyp size in patients with CRSwNP, the beneficial effects are short lived once discontinued (8–12 weeks when used in combination with topical intranasal corticosteroids) (14, 48). Current guidelines restrict the use of SGC to short courses to manage CRSwNP exacerbations. SGC maintenance treatment is rarely prescribed for this pathology (9, 48, 49).

The *Global Initiative for Asthma* for the management and prevention of asthma recommends using only short-courses of SGC to manage severe asthma exacerbations in adults: 40–50 mg/day usually for 5–7 days (Evidence Level D) (32). An Expert Consensus on the Tapering of Oral Corticosteroids for the Treatment of Asthma involving 131 international experts agreed that 5–7 days should be the maximal duration for a short course of SGC for treatment of an exacerbation and that the optimal dosage of a short course of SGC should be 0.5 mg/kg/day (33). In situations where SGC maintenance treatment is necessary, they considered ≤ 5 mg/day to be an acceptable maximum threshold. The magnitude of dose reduction and speed of SGC tapering needs to be individualized for each patient (33).

### 3.2 Use of biologics as an alternative to SGC in eosinophil-related diseases

The benefits of SGC need to be balanced against SGC-associated AEs, which place a large burden on patients and healthcare systems (37, 50). Biologics allow discontinuation or significant dose reduction of SGC and improve SGC stewardship (11).

Monoclonal antibodies against IL-5 (mepolizumab, reslizumab) or the IL-5 receptor (benralizumab) have been approved by the Food and Drug Administration (FDA) and the European Medicines Agency (EMA) for the treatment of eosinophilic asthma. Mepolizumab has also been approved in the USA and the EU for EGPA, HES and CRSwNP. These biological agents reduce the risk of severe exacerbations and are effective glucocorticoid-sparing agents in patients with eosinophil-related diseases (51–53).

#### 3.2.1 Biologics for systemic eosinophilic diseases: EGPA and HES

Biologics are generally considered in non-severe patients with a maintenance dose of prednisone or equivalent that cannot be reduced below 7.5 mg/day OR who have two or more relapses/exacerbations per year requiring higher doses of SGC to induce remission.

In EGPA, the severity of the disease (renal involvement, alveolar hemorrhage, infiltrative cardiomyopathy, mesenteric ischemia, multiple mononeuritis or central nervous involvement) and the presence of poor prognostic factors, such as age > 65 years, serum creatinine > 150 μmol/L, and/or no ear, nose and throat involvement, condition the use of SGC and conventional immunosuppressants such as methotrexate, azathioprine, cyclophosphamide or mycophenolate mofetil (6, 7, 12).

For patients in remission maintenance with non severe-EGPA, 300 mg of mepolizumab every 4 weeks can be added to the standard of care to decrease the risk of flare ups and reduce the use of glucocorticoids (25, 54). The results of a multicenter, double-blind, parallel-group, phase 3 trial, showed that mepolizumab resulted in a significantly higher proportion of participants in remission and a longer remission duration than placebo, allowing to reduce glucocorticoid dosage (25). Results from a retrospective study of a large European EGPA cohort suggested that similar complete response rates could be achieved with 100 mg of mepolizumab every 4 weeks (55). The experts agreed that in patients treated with a combination of SGC, immunosuppressants and mepolizumab, SGC should be withdrawn first and then the immunosuppressants so, if possible, patients remain only on mepolizumab.

For inducing remission in patients with EGPA, there is currently a trial underway that is comparing a mepolizumab-based regimen to conventional therapeutic strategy (SGC alone or in combination with conventional immunosuppressive agents) (NCT05030155).

The use of the anti-CD20 monoclonal antibody rituximab is conditionally recommended in current guidelines to induce remission in patients with active, severe EGPA (7, 40). Ongoing trials will test the potential clinical benefits of using rituximab, reslizumab and benralizumab for the treatment of EGPA (56).

Using biologics early in patients with higher risk of developing SGC-related AEs, such as those with hypertension, diabetes, heart failure, glaucoma, low bone density or immunodeficiency could help to accelerate the withdrawal of immunosuppressants and SGC and thus, prevent these SGC-related and disease-related risks.

In HES, signs of organ damage and/or significant increase of the eosinophil count with a prednisone dose > 10 mg daily is an indication for the addition of other agents (8). While 300 mg mepolizumab has been approved for patients with idiopathic HES and has shown efficacy in decreasing the risk of flares as well as a steroid-sparing therapy (57, 58), other anti-IL-5/anti-IL-5 receptor and anti-CD52 antibody approaches for the treatment of HES remain investigational (8, 59, 60). A phase 3 trial of benralizumab (NCT04191304) is ongoing. The factors predicting response to a given therapy are still largely unknown (61).

Several genetic abnormalities define myeloid/lymphoid neoplasms with eosinophilia and tyrosine kinase gene fusions (62). Patients with these abnormalities are not considered to have HES and may benefit from targeted therapies. Patients with *FIP1L1- PDGFRA* or *PDGFRB* rearrangements, should be prescribed imatinib mesylate (100mg and 400 mg daily, respectively) as first-line therapy (61).

#### 3.2.2 For respiratory eosinophilic diseases: CRSwNP and SEA-EP

In CRSwNP patients, the experts would consider using a biologic in those who continue to experience severe symptoms after their first surgery. They also agreed with the EPOS 2020 guidelines stating that in patients with bilateral polyps who are not candidates for surgery, at least three of the following criteria should be met for starting a biological: 1) Evidence of type 2 disease; 2) need for at least two courses of SGC per year or continuous use (>3 months) of low-dose SGC or contraindication to SGC; 3) significantly impaired quality of life (SNOT-22 ≥40); 4) anosmia on smell test; 5) diagnosis of comorbid asthma (not necessarily severe) (9).

Up to 60% of patients with CRSwNP experience disease recurrence after surgery (63, 64) and previous treatment with SGC together with other comorbidities is a predictive factor for revision surgeries (65). Operated patients are likely to respond faster to biological treatment due to reduced inflammation (66–68). Furthermore, the use of a biologic after first surgery may avert the need for repeated or revision surgeries (69).

Both dupilumab and mepolizumab have been shown to reduce symptoms, improve quality of life, and reduce the need for SGC and surgery over the course of 1 year of treatment versus placebo (70). Further research into the efficacy of biologics in relation to the timing of surgery, combination approaches, their long-term safety and cost-effectiveness, in the context of patient preferences and goals is required (68).

The use of biologics in severe asthma is well defined in clinical guidelines for managing the disease. GINA and Spanish Asthma Management Guidelines (71) now recommend biological agents as a preferred treatment choice over the use of SGC. Thus, biologics are recommended before chronic SGC in uncontrolled patients (72). Despite the proven reduction in exacerbation rates and SGC-sparing effects in real-life studies (73, 74), biologics are still under-prescribed in most regions in Spain (75, 76).

To target patients who are most likely to benefit from biologics, healthcare professionals should assess: adherence to inhaled treatment, comorbidities (such as CRSwNP, atopic dermatitis, food allergy, sleep apnea, gastroesophageal reflux disease and psychiatric conditions including depression and anxiety), presence of other diseases (especially those that mimic asthma-like symptoms, mainly but not exclusively chronic obstructive pulmonary disease (COPD), heart failure and inducible laryngeal obstruction) and other individual risk factors that might be corrected (such as smoking, allergen exposure, infections, obesity, among others). Ideally, all these factors should be rapidly identified and optimally addressed before considering a biologic.

There is an urgent need for standardized guidelines on the implementation of SGC weaning protocols following biologic initiation in patients with severe asthma (77).

### 3.3 Measures to prevent AEs

The potential benefits of SGC therapy must be weighed against the risk of AEs in every individual patient. Patients on SGC should be followed carefully to prevent AEs. Early detection of AEs is important in the treatment and management of SGC-related complications (78).

All SGC prescriptions should be discussed among specialists treating individual patients to ensure awareness of other concomitant conditions that may also require SGC (in which case the cumulative dose should be calculated) and also of concomitant medications that could reduce the clearance of SGC by interfering with cytochrome P450 3A4 activity.

In asthma, even a short burst of SGC can be associated with AEs and each SGC prescription results in a cumulative burden, regardless of the dose and duration (79, 80). The risk of adverse outcomes is evident and statistically significant with cumulative SGC exposure of 1 to 2.5 g per year (34).

In the case of respiratory eosinophilic disease, the intake of SGC is sometimes subject to patient self-administration and management, based on previous experiences, and sometimes even according to a self-management written action plan agreed with the treating physician that patients may interpret subjectively and/or not further consult with their doctor in case of rapid improvement. Thus, the cumulative dose may easily exceed the recommended maximum. To avoid this, the experts suggest prescribing smaller amounts of medication at a time and educating patients about the risk of SGC-related AEs (47).

The experts recommend asking patients with severe CRSwNP or SA-EP about self-medications with SGC at each follow-up visit, and developing a written action plan for each patient that clearly indicates what to do in the event of an exacerbation and when to consult a doctor.

The thresholds per year in respiratory disease and per day in systemic disease at which the risks of developing SGC-related AEs drastically outweigh the benefits still need to be established and adjusted for age. Prompt administration of SGC-sparing agents could ensure greater therapeutic success and prevent the risk of developing SGC-related AEs.

Different specialties follow different protocols to prevent SGC-related AEs. Although there are no reports indicating that the AEs of SGC are different between systemic and respiratory eosinophilic diseases, the protocols to prevent AEs are better established for systemic eosinophilic diseases. Patients on recurrent or maintenance SGC require more follow-ups to monitor their body mass index, blood pressure, intraocular pressure, cardiovascular risk factors and glycaemia (81). In addition, to prevent osteoporosis and risk of severe infection or reactivation of chronic infections, the authors recommend following guidelines such as The Spanish Society of Rheumatology’s guidelines for managing patients treated with long-term SGC or the Recommendations for Prevention of Infection in Systemic Autoimmune Rheumatic Diseases (16, 82) (**Table 2**).

**Table 2:** Measures to monitor for SGC-related AEs in patients with eosinophil-related diseases.

AE	Measure	Frequency
***Physical***	Weight	Every check up
	BMI	
	Blood pressure	
	Blood count (every 6-12 months)	
***HPA-axis functioning***	Biochemical testing of the HPA axis (morning cortisol, ITT, ACTH stimulation)	If adrenal suppression suspected
***Dyslipidemia and CV risk***	Assess lipid profile	At baseline, 1 month after SGC initiation and then every 6–12 months
	Assess 10-year CV risk using FRS	Every 5 years
***Hyperglycemia/Diabetes***	Monitor glucose parameters	For at least 48 hours after SGC initiation, then every 3–6 months for first year; annually thereafter
***Ocular system***	Refer for examination by ophthalmologist	Annually
***Bone health and osteoporosis***	BMD	At the start of treatment and 1-year post SGC initiation. If stable, assess every 2-3 years. If decreased, assess annually
***Severe infection or reactivation of chronic infection***	Prescribe trimethoprim-sulfamethoxazole as prophylaxis against *Pneumocystis jirovecii* and consider vaccinations against Hepatitis B, *Streptococcus pneumoniae*, Herpes Zoster, Human papillomavirus and influenza viruses. Screen for latent tuberculosis.	Before starting treatment

Up to 40% of individuals taking glucocorticoids develop bone loss over time (83). The Spanish Society of Rheumatology recommends starting pharmacological treatment to prevent osteoporosis in patients receiving ≥ 30 mg/day of prednisone for more than 3 months; in postmenopausal women and men > 50 years of age with low bone mineral density (BMD) receiving doses of prednisone ≥ 5 mg/day for more than 3 months; and in premenopausal women and men < 50 years of age with low BMD receiving doses of prednisone ≥ 7.5 mg/day for more than 3 months (82).

Current guidelines recommend using trimethoprim-sulfamethoxazole as prophylaxis against *Pneumocystis jirovecii* in patients receiving ≥ 20 mg/day of prednisone; and starting concomitant treatment with antivirals in patients with chronic HBV infection and who are going to receive prednisone or equivalent ≥ 20 mg/day for 4 weeks.

The Glucocorticoid Toxicity Index (GTI) is now in use across a number of diseases, including ANCA-associated vasculitis. The GTI aims to evaluate SGC toxicity and how it changes over time following the introduction of SGC-sparing agents (84, 85). Further knowledge of what constitutes a significant toxicity change could be used as a measure of efficacy of SCS-sparing agents in individual patients, aiding head-to-head comparisons between different drugs.

## 4 Conclusions

Patients with eosinophil-related diseases require a well-structured and multifaceted treatment approach that considers the presence of co-morbidities, as these can limit the response to treatment or cause similar symptoms or exacerbations.

Treatment recommendations should be interpreted considering the individual patient’s preferences, personal and clinical circumstances, and expectations.

Awareness of the potentially harmful effects of SGC, regardless of the dose, duration or frequency of administration, still needs to be emphasized among healthcare professionals that manage eosinophil-related diseases.

Biologics represent an alternative, well-tolerated option for patients with eosinophilic diseases that allow healthcare professionals to reduce or suspend SGC and avoid the risk of SGC-related AEs. The efficacy and safety of anti-IL-5 therapies, largely derived from their selectivity, have considerably advanced the management of eosinophil-related diseases. An earlier implementation of biological therapies in the course of the disease might help prevent the damage associated with the medium and long-term use of SGC.

## Author contributions

VP: Data curation, Investigation, Writing – review & editing. IB: Data curation, Investigation, Writing – review & editing. MJR: Data curation, Investigation, Writing – review & editing. GE-F: Data curation, Investigation, Writing – review & editing. RSL: Data curation, Investigation, Writing – review & editing. JMH-R: Data curation, Investigation, Writing – review & editing. EM: Data curation, Investigation, Writing – review & editing. AC-L: Data curation, Investigation, Writing – review & editing. JLIA: Data curation, Investigation, Writing – review & editing. MSDS: Data curation, Investigation, Writing – review & editing. JM-S: Data curation, Investigation, Writing – review & editing. BA-M: Data curation, Investigation, Writing – review & editing. DB-C: Data curation, Investigation, Writing – review & editing. ALM: Data curation, Investigation, Writing – review & editing. ÍR-F: Data curation, Investigation, Writing – review & editing.
