# Supplementary material for: Expert consensus on the use of systemic glucocorticoids for managing eosinophil-related diseases
Source: Front Immunol. 2024 Jan 5;14:1310211. doi: 10.3389/fimmu.2023.1310211 (PMC10796442; doi:10.3389/fimmu.2023.1310211)
Supplement: Supplementary file 1 [file DataSheet_1.docx]

**Bibliographic search – Respiratory diseases**

**Timeframe:** 2007-2022

**Keywords:** oral glucocorticoids, systemic glucocorticoids, corticosteroids, eosinophils, eosinophilic diseases, chronic rhinosinusitis, nasal polyps, polyposis, severe asthma

**TREATMENT WITH ORAL GLUCOCORTICOIDS IN:**

**CHRONIC RHINOSINUSITIS WITH NASAL POLYPS**

De Corso E, et al. Survey on Use of Local and Systemic Corticosteroids in the Management of Chronic Rhinosinusitis with Nasal Polyps: Identification of Unmet Clinical Needs. J Pers Med. 2022 May 29;12(6):897. doi: 10.3390/jpm12060897.

Fokkens WJ, Lund VJ, Hopkins C, Hellings PW, Kern R, et al. European Position Paper on Rhinosinusitis and Nasal Polyps 2020. Rhinology. 2020 Feb 20;58(Suppl S29):1-464. doi: 10.4193/Rhin20.600. PMID: 32077450.

Gelardi M, Barbara F, Covelli I, Damiani MA, Plantone F, Notarnicola A, Moretti B, Quaranta N, Ciprandi G. Long-Term Therapy with Corticosteroids in Nasal Polyposis: A Bone Metabolism Assessment. Indian J Otolaryngol Head Neck Surg. 2019 Nov;71(Suppl 3):2050-2056. doi: 10.1007/s12070-018-1466-5. Epub 2018 Aug 1. PMID: 31763293; PMCID: PMC6848363.

Head K, Chong LY, Hopkins C, Philpott C, Burton MJ, Schilder AG. Short-course oral steroids alone for chronic rhinosinusitis. Cochrane Database Syst Rev. 2016 Apr 26;4(4):CD011991. doi: 10.1002/14651858.CD011991.pub2. PMID: 27113367; PMCID: PMC8504433.

Head K, Chong LY, Hopkins C, Philpott C, Schilder AG, Burton MJ. Short-course oral steroids as an adjunct therapy for chronic rhinosinusitis. Cochrane Database Syst Rev. 2016 Apr 26;4(4):CD011992. doi: 10.1002/14651858.CD011992.pub2. PMID: 27115214; PMCID: PMC8763342.

Howard BE, Lal D. Oral steroid therapy in chronic rhinosinusitis with and without nasal polyposis. Curr Allergy Asthma Rep. 2013 Apr;13(2):236-43. doi: 10.1007/s11882-012-0329-5. PMID: 23225105.

Hox V, Lourijsen E, Jordens A, Aasbjerg K, Agache I, Alobid I, Bachert C, Boussery K, Campo P, Fokkens W, Hellings P, Hopkins C, Klimek L, Mäkelä M, Mösges R, Mullol J, Pujols L, Rondon C, Rudenko M, Toppila-Salmi S, Scadding G, Scheire S, Tomazic PV, Van Zele T, Wagemann M, van Boven JFM, Gevaert P. Benefits and harm of systemic steroids for short- and long-term use in rhinitis and rhinosinusitis: an EAACI position paper. Clin Transl Allergy. 2020 Jan 3;10:1. doi: 10.1186/s13601-019-0303-6. Erratum in: Clin Transl Allergy. 2020 Sep 28;10:38. PMID: 31908763; PMCID: PMC6941282.

Poetker DM, Jakubowski LA, Lal D, Hwang PH, Wright ED, Smith TL. Oral corticosteroids in the management of adult chronic rhinosinusitis with and without nasal polyps: an evidence-based review with recommendations. Int Forum Allergy Rhinol. 2013 Feb;3(2):104-20. doi: 10.1002/alr.21072. Epub 2012 Aug 7. PMID: 22887970.

Sahlstrand-Johnson P, Holmström M, Ehnhage A. Does the oral steroid treatment of patients with nasal polyposis cause osteopenia or osteoporosis? Clin Otolaryngol. 2019 Nov;44(6):1011-1016. doi: 10.1111/coa.13431. Epub 2019 Oct 27. PMID: 31529761.

Scadding GK, Durham SR, Mirakian R, Jones NS, Drake-Lee AB, Ryan D, Dixon TA, Huber PA, Nasser SM; British Society for Allergy and Clinical Immunology. BSACI guidelines for the management of rhinosinusitis and nasal polyposis. Clin Exp Allergy. 2008 Feb;38(2):260-75. doi: 10.1111/j.1365-2222.2007.02889.x. Epub 2007 Dec 20. PMID: 18167126.

Scott JR, Ernst HM, Rotenberg BW, Rudmik L, Sowerby LJ. Oral corticosteroid prescribing habits for rhinosinusitis: The American Rhinologic Society membership. Am J Rhinol Allergy. 2017 Jan 1;31(1):22-26. doi: 10.2500/ajra.2017.31.4396. PMID: 28234148.

Winblad L, Larsen CG, Håkansson K, Abrahamsen B, von Buchwald C. The risk of osteoporosis in oral steroid treatment for nasal polyposis: a systematic review. Rhinology. 2017 Sep 1;55(3):195-201. doi: 10.4193/Rhino15.367. PMID: 28492609.

Xu Z, Luo X, Xu L, Deng J, Gao W, Jiang L, Huang Z, Shi J, Lai Y. Effect of short-course glucocorticoid application on patients with chronic rhinosinusitis with nasal polyps. World Allergy Organ J. 2020 Jun 11;13(6):100131. doi: 10.1016/j.waojou.2020.100131. PMID: 32566071; PMCID: PMC7300158.

Zhang Y, Lou H, Wang Y, Li Y, Zhang L, Wang C. Comparison of Corticosteroids by 3 Approaches to the Treatment of Chronic Rhinosinusitis With Nasal Polyps. Allergy Asthma Immunol Res. 2019 Jul;11(4):482-497. doi: 10.4168/aair.2019.11.4.482. PMID: 31172717; PMCID: PMC6557780.

**SEVERE ASTHMA**

Barry LE, Sweeney J, O'Neill C, Price D, Heaney LG. The cost of systemic corticosteroid-induced morbidity in severe asthma: a health economic analysis. Respir Res. 2017 Jun 26;18(1):129. doi: 10.1186/s12931-017-0614-x. PMID: 28651591; PMCID: PMC5485660.

Blakey J, Chung LP, McDonald VM, Ruane L, Gornall J, Barton C, Bosnic-Anticevich S, Harrington J, Hew M, Holland AE, Hopkins T, Jayaram L, Reddel H, Upham JW, Gibson PG, Bardin P. Oral corticosteroids stewardship for asthma in adults and adolescents: A position paper from the Thoracic Society of Australia and New Zealand. Respirology. 2021 Dec;26(12):1112-1130. doi: 10.1111/resp.14147. Epub 2021 Sep 29. PMID: 34587348; PMCID: PMC9291960.

Bleecker ER, Menzies-Gow AN, Price DB, Bourdin A, Sweet S, Martin AL, Alacqua M, Tran TN. Systematic Literature Review of Systemic Corticosteroid Use for Asthma Management. Am J Respir Crit Care Med. 2020 Feb 1;201(3):276-293. doi: 10.1164/rccm.201904-0903SO. PMID: 31525297; PMCID: PMC6999108.

Bourdin A, Husereau D, Molinari N, Golam S, Siddiqui MK, Lindner L, Xu X. Matching-adjusted comparison of oral corticosteroid reduction in asthma: Systematic review of biologics. Clin Exp Allergy. 2020 Apr;50(4):442-452. doi: 10.1111/cea.13561. Epub 2020 Mar 20. PMID: 31943429; PMCID: PMC7204869.

Cataldo D, Louis R, Michils A, Peché R, Pilette C, Schleich F, Ninane V, Hanon S. Severe asthma: oral corticosteroid alternatives and the need for optimal referral pathways. J Asthma. 2021 Apr;58(4):448-458. doi: 10.1080/02770903.2019.1705335. Epub 2020 Jan 11. PMID: 31928102.

Chalitsios CV, Shaw DE, McKeever TM. Risk of osteoporosis and fragility fractures in asthma due to oral and inhaled corticosteroids: two population-based nested case-control studies. Thorax. 2021 Jan;76(1):21-28. doi: 10.1136/thoraxjnl-2020-215664. Epub 2020 Oct 21. PMID: 33087546.

Chung LP, Upham JW, Bardin PG, Hew M. Rational oral corticosteroid use in adult severe asthma: A narrative review. Respirology. 2020 Feb;25(2):161-172. doi: 10.1111/resp.13730. Epub 2019 Nov 12. PMID: 31713955; PMCID: PMC7027745.

Dalal AA, Duh MS, Gozalo L, Robitaille MN, Albers F, Yancey S, Ortega H, Forshag M, Lin X, Lefebvre P. Dose-Response Relationship Between Long-Term Systemic Corticosteroid Use and Related Complications in Patients with Severe Asthma. J Manag Care Spec Pharm. 2016 Jul;22(7):833-47. doi: 10.18553/jmcp.2016.22.7.833. PMID: 27348284.

Gurnell M, Heaney LG, Price D, Menzies-Gow A. Long-term corticosteroid use, adrenal insufficiency and the need for steroid-sparing treatment in adult severe asthma. J Intern Med. 2021 Aug;290(2):240-256. doi: 10.1111/joim.13273. Epub 2021 Apr 1. PMID: 33598993; PMCID: PMC8360169.

Korn S, Howarth P, Smith SG, Price RG, Yancey SW, Prazma CM, Bel EH. Development of methodology for assessing steroid-tapering in clinical trials for biologics in asthma. Respir Res. 2022 Mar 4;23(1):45. doi: 10.1186/s12931-022-01959-1. PMID: 35246123; PMCID: PMC8896284.

Kwon JW, Kim MA, Sim DW, Lee HY, Rhee CK, Yang MS, Shim JS, Kim MH, Kim SR, Park CS, Kim BK, Kang SY, Choi GS, Lee H, Jang AS, Kim SH; Korean Academy of Asthma, Allergy, and Clinical Immunology (KAAACI), the Working Group on Severe Asthma. Prescription Patterns of Oral Corticosteroids for Asthma Treatment and Related Asthma Phenotypes in University Hospitals in Korea. Allergy Asthma Immunol Res. 2022 May;14(3):300-313. doi: 10.4168/aair.2022.14.3.300. PMID: 35557495; PMCID: PMC9110914.

Lee JH, Kim HJ, Park CS, Park SY, Park SY, Lee H, Kim SH, Cho YS; Working Group on Severe Asthma, the Korean Academy of Asthma, Allergy and Clinical Immunology. Clinical Characteristics and Disease Burden of Severe Asthma According to Oral Corticosteroid Dependence: Real-World Assessment From the Korean Severe Asthma Registry (KoSAR). Allergy Asthma Immunol Res. 2022 Jul;14(4):412-423. doi: 10.4168/aair.2022.14.4.412. PMID: 35837824; PMCID: PMC9293595.

Price D, Castro M, Bourdin A, Fucile S, Altman P. Short-course systemic corticosteroids in asthma: striking the balance between efficacy and safety. Eur Respir Rev. 2020 Apr 3;29(155):190151. doi: 10.1183/16000617.0151-2019. PMID: 32245768.

Price DB, Trudo F, Voorham J, Xu X, Kerkhof M, Ling Zhi Jie J, Tran TN. Adverse outcomes from initiation of systemic corticosteroids for asthma: long-term observational study. J Asthma Allergy. 2018 Aug 29;11:193-204. doi: 10.2147/JAA.S176026. PMID: 30214247; PMCID: PMC6121746.

Rice JB, White AG, Scarpati LM, Wan G, Nelson WW. Long-term Systemic Corticosteroid Exposure: A Systematic Literature Review. Clin Ther. 2017 Nov;39(11):2216-2229. doi: 10.1016/j.clinthera.2017.09.011. Epub 2017 Oct 19. PMID: 29055500.

Sullivan PW, Ghushchyan VH, Globe G, Schatz M. Oral corticosteroid exposure and adverse effects in asthmatic patients. J Allergy Clin Immunol. 2018 Jan;141(1):110-116.e7. doi: 10.1016/j.jaci.2017.04.009. Epub 2017 Apr 27. PMID: 28456623.

Sweeney J, Patterson CC, Menzies-Gow A, Niven RM, Mansur AH, Bucknall C, Chaudhuri R, Price D, Brightling CE, Heaney LG; British Thoracic Society Difficult Asthma Network. Comorbidity in severe asthma requiring systemic corticosteroid therapy: cross-sectional data from the Optimum Patient Care Research Database and the British Thoracic Difficult Asthma Registry. Thorax. 2016 Apr;71(4):339-46. doi: 10.1136/thoraxjnl-2015-207630. Epub 2016 Jan 27. PMID: 26819354.

Tran TN, King E, Sarkar R, Nan C, Rubino A, O'Leary C, Muzwidzwa R, Belton L, Quint JK. Oral corticosteroid prescription patterns for asthma in France, Germany, Italy and the UK. Eur Respir J. 2020 Jun 4;55(6):1902363. doi: 10.1183/13993003.02363-2019. PMID: 32165402; PMCID: PMC7270349.

Volmer T, Effenberger T, Trautner C, Buhl R. Consequences of long-term oral corticosteroid therapy and its side-effects in severe asthma in adults: a focused review of the impact data in the literature. Eur Respir J. 2018 Oct 25;52(4):1800703. doi: 10.1183/13993003.00703-2018. PMID: 30190274.

Zazzali JL, Broder MS, Omachi TA, Chang E, Sun GH, Raimundo K. Risk of corticosteroid-related adverse events in asthma patients with high oral corticosteroid use. Allergy Asthma Proc. 2015 Jul-Aug;36(4):268-74. doi: 10.2500/aap.2015.36.3863. PMID: 26108084.

**Bibliographic search – Systemic diseases**

**Timeframe:** 2007-2022

**Keywords:** oral glucocorticoids, systemic glucocorticoids, corticosteroids, eosinophils, eosinophilic diseases, hypereosinophilic syndrome, eosinophilic granulomatosis with polyangiitis, Churg-Strauss syndrome

**TREATMENT WITH ORAL GLUCOCORTICOIDS IN:**

**EOSINOPHILIC GRANULOMATOSIS WITH POLYANGIITIS**

Bell CF, Blauer-Peterson C, Mao J. Burden of illness and costs associated with eosinophilic granulomatosis with polyangiitis: evidence from a managed care database in the United States. J Manag Care Spec Pharm. 2021 Sep;27(9):1249-1259. doi: 10.18553/jmcp.2021.21002. Epub 2021 Jun 24. PMID: 34165321.

Box CD, Cronin O, Hauser B. The Impact of High Dose Glucocorticoids on Bone Health and Fracture Risk in Systemic Vasculitides. Front Endocrinol (Lausanne). 2022 Feb 16;13:806361. doi: 10.3389/fendo.2022.806361. PMID: 35250864; PMCID: PMC8889574.

Canzian A, Venhoff N, Urban ML, Sartorelli S, Ruppert AM, Groh M, et al. French Vasculitis Study Group and the European EGPA Study Group. Use of Biologics to Treat Relapsing and/or Refractory Eosinophilic Granulomatosis With Polyangiitis: Data From a European Collaborative Study. Arthritis Rheumatol. 2021 Mar;73(3):498-503. doi: 10.1002/art.41534. Epub 2021 Jan 23. PMID: 33001543.

Comarmond C, Pagnoux C, Khellaf M, Cordier JF, Hamidou M, Viallard JF, Maurier F, Jouneau S, Bienvenu B, Puéchal X, Aumaître O, Le Guenno G, Le Quellec A, Cevallos R, Fain O, Godeau B, Seror R, Dunogué B, Mahr A, Guilpain P, Cohen P, Aouba A, Mouthon L, Guillevin L; French Vasculitis Study Group. Eosinophilic granulomatosis with polyangiitis (Churg-Strauss): clinical characteristics and long-term followup of the 383 patients enrolled in the French Vasculitis Study Group cohort. Arthritis Rheum. 2013 Jan;65(1):270-81. doi: 10.1002/art.37721. PMID: 23044708.

Doubelt I, Cuthbertson D, Carette S, Chung SA, Forbess LJ, Khalidi NA, Koening CL, Langford C, McAlear CA, Moreland LW, Monach PA, Seo P, Specks U, Spiera RF, Springer JM, Sreih AG, Warrington KJ, Merkel PA, Pagnoux C; Vasculitis Clinical Research Consortium. Clinical Manifestations and Long-Term Outcomes of Eosinophilic Granulomatosis With Polyangiitis in North America. ACR Open Rheumatol. 2021 Jun;3(6):404-412. doi: 10.1002/acr2.11263. Epub 2021 May 25. PMID: 34032390; PMCID: PMC8207688.

Durel CA, Berthiller J, Caboni S, Jayne D, Ninet J, Hot A. Long-Term Followup of a Multicenter Cohort of 101 Patients With Eosinophilic Granulomatosis With Polyangiitis (Churg-Strauss). Arthritis Care Res (Hoboken). 2016 Mar;68(3):374-87. doi: 10.1002/acr.22686. PMID: 26315340.

Gokhale M, Bell CF, Doyle S, Fairburn-Beech J, Steinfeld J, Van Dyke MK. Prevalence of Eosinophilic Granulomatosis With Polyangiitis and Associated Health Care Utilization Among Patients With Concomitant Asthma in US Commercial Claims Database. J Clin Rheumatol. 2021 Apr 1;27(3):107-113. doi: 10.1097/RHU.0000000000001198. PMID: 31693654; PMCID: PMC7996234.

Groh M, Pagnoux C, Baldini C, Bel E, Bottero P, Cottin V, Dalhoff K, Dunogué B, Gross W, Holle J, Humbert M, Jayne D, Jennette JC, Lazor R, Mahr A, Merkel PA, Mouthon L, Sinico RA, Specks U, Vaglio A, Wechsler ME, Cordier JF, Guillevin L. Eosinophilic granulomatosis with polyangiitis (Churg-Strauss) (EGPA) Consensus Task Force recommendations for evaluation and management. Eur J Intern Med. 2015 Sep;26(7):545-53. doi: 10.1016/j.ejim.2015.04.022. Epub 2015 May 9. PMID: 25971154.

Raffray L, Guillevin L. Updates for the treatment of EGPA. Presse Med. 2020 Oct;49(3):104036. doi: 10.1016/j.lpm.2020.104036. Epub 2020 Jul 8. PMID: 32652104.

Sada KE, Kojo Y, Fairburn-Beech J, Sato K, Akiyama S, Van Dyke MK, Mukai I. The prevalence, burden of disease, and healthcare utilization of patients with eosinophilic granulomatosis with polyangiitis in Japan: a retrospective, descriptive cohort claims database study. Mod Rheumatol. 2022 Feb 28;32(2):380-386. doi: 10.1093/mr/roab007. PMID: 34894250.

Silva M, Roufosse F. Oral Corticosteroid Use for the Treatment of Chronic Eosinophilic Disease: A Patient's and His Physician's Experience. Adv Ther. 2019 Oct;36(10):2558-2566. doi: 10.1007/s12325-019-01070-w. Epub 2019 Aug 31. PMID: 31473972; PMCID: PMC6822817.

Terrier B, Darbon R, Durel CA, Hachulla E, Karras A, Maillard H, Papo T, Puechal X, Pugnet G, Quemeneur T, Samson M, Taille C, Guillevin L; Collaborators. French recommendations for the management of systemic necrotizing vasculitides (polyarteritis nodosa and ANCA-associated vasculitides). Orphanet J Rare Dis. 2020 Dec 29;15(Suppl 2):351. doi: 10.1186/s13023-020-01621-3. Erratum in: Orphanet J Rare Dis. 2021 Apr 6;16(1):155. PMID: 33372616; PMCID: PMC7771069.

**HYPEREOSINOPHILIC SYNDROME**

Alves Júnior JM, Prota FE, Villagelin D, Bley F, Bernardo WM. Mepolizumab in Hypereosinophilic Syndrome: A Systematic Review and Meta-analysis. Clinics (Sao Paulo). 2021 Oct 11;76:e3271. doi: 10.6061/clinics/2021/e3271. PMID: 34644737; PMCID: PMC8478134.

Chen MM, Roufosse F, Wang SA, Verstovsek S, Durrani SR, Rothenberg ME, Pongdee T, Butterfield J, Lax T, Wechsler ME, Stein ML, Ogbogu PU, Kahwash BM, Mathur SK, Simon D, Akuthota P, Holland N, Wetzler L, Ware JM, Guo C, Fay MP, Khoury P, Klion AD, Bochner BS. An International, Retrospective Study of Off-Label Biologic Use in the Treatment of Hypereosinophilic Syndromes. J Allergy Clin Immunol Pract. 2022 May;10(5):1217-1228.e3. doi: 10.1016/j.jaip.2022.02.006. Epub 2022 Feb 15. PMID: 35181548; PMCID: PMC9086180.

Helbig G, Kyrcz-Krzemień S. Diagnostic and therapeutic management in patients with hypereosinophilic syndromes. Pol Arch Med Wewn. 2011 Jan-Feb;121(1-2):44-52. PMID: 21346698.

Khoury P, Abiodun AO, Holland-Thomas N, Fay MP, Klion AD. Hypereosinophilic Syndrome Subtype Predicts Responsiveness to Glucocorticoids. J Allergy Clin Immunol Pract. 2018 Jan-Feb;6(1):190-195. doi: 10.1016/j.jaip.2017.06.006. Epub 2017 Jul 27. PMID: 28757367; PMCID: PMC5760470.

Ogbogu PU, Bochner BS, Butterfield JH, Gleich GJ, Huss-Marp J, Kahn JE, Leiferman KM, Nutman TB, Pfab F, Ring J, Rothenberg ME, Roufosse F, Sajous MH, Sheikh J, Simon D, Simon HU, Stein ML, Wardlaw A, Weller PF, Klion AD. Hypereosinophilic syndrome: a multicenter, retrospective analysis of clinical characteristics and response to therapy. J Allergy Clin Immunol. 2009 Dec;124(6):1319-25.e3. doi: 10.1016/j.jaci.2009.09.022. PMID: 19910029; PMCID: PMC2829669.

Pavord ID, Bel EH, Bourdin A, Chan R, Han JK, Keene ON, Liu MC, Martin N, Papi A, Roufosse F, Steinfeld J, Wechsler ME, Yancey SW. From DREAM to REALITI-A and beyond: Mepolizumab for the treatment of eosinophil-driven diseases. Allergy. 2022 Mar;77(3):778-797. doi: 10.1111/all.15056. Epub 2021 Sep 16. PMID: 34402066; PMCID: PMC9293125.

Roufosse F, de Lavareille A, Schandené L, Cogan E, Georgelas A, Wagner L, Xi L, Raffeld M, Goldman M, Gleich GJ, Klion A. Mepolizumab as a corticosteroid-sparing agent in lymphocytic variant hypereosinophilic syndrome. J Allergy Clin Immunol. 2010 Oct;126(4):828-835.e3. doi: 10.1016/j.jaci.2010.06.049. PMID: 20810155; PMCID: PMC2950246.

Schwartz LB, Sheikh J, Singh A. Current strategies in the management of hypereosinophilic syndrome, including mepolizumab. Curr Med Res Opin. 2010 Aug;26(8):1933-46. doi: 10.1185/03007995.2010.493132. PMID: 20565230.

Shomali W, Gotlib J. World Health Organization-defined eosinophilic disorders: 2022 update on diagnosis, risk stratification, and management. Am J Hematol. 2022 Jan 1;97(1):129-148. doi: 10.1002/ajh.26352. Epub 2021 Oct 8. PMID: 34533850.

Stokes K, Yoon P, Makiya M, Gebreegziabher M, Holland-Thomas N, Ware J, Wetzler L, Khoury P, Klion AD. Mechanisms of glucocorticoid resistance in hypereosinophilic syndromes. Clin Exp Allergy. 2019 Dec;49(12):1598-1604. doi: 10.1111/cea.13509. Epub 2019 Oct 27. PMID: 31657082; PMCID: PMC6910955.
